# Intractable Persistent Direction-Changing Geotropic Nystagmus Improved by Lateral Semicircular Canal Plugging

**DOI:** 10.1155/2015/192764

**Published:** 2015-01-01

**Authors:** Toru Seo, Kazuya Saito, Katsumi Doi

**Affiliations:** Department of Otolaryngology, Kinki University Faculty of Medicine, 377-2 Ohno-higashi, Osakasayama, Osaka 589-8511, Japan

## Abstract

Antigravitational deviation of the cupula of the lateral semicircular canal, which is also called light cupula, evokes persistent direction-changing geotropic nystagmus with a neutral point. No intractable cases of this condition have been reported. In our case, a 67-year-old man complained of positional vertigo 3 months after developing idiopathic sudden hearing loss in the right ear with vertigo. He showed a persistent direction-changing geotropic nystagmus with a leftward beating nystagmus in the supine position. The nystagmus resolved when his head was turned approximately 30° to the right. He was diagnosed with light cupula of the right lateral semicircular canal and was subsequently treated with an antivertiginous agent. However, his symptoms and positional nystagmus did not improve, so the right lateral semicircular canal was plugged by surgery. One month after surgery, his positional vertigo and nystagmus were completely resolved. We speculated that the cause of the patient's intractable light cupula was an enlarged cupula caused by his idiopathic sudden hearing loss.

## 1. Introduction

Benign paroxysmal positional vertigo (BPPV) is the most common disorder associated with vertigo. The clinical features of BPPV were first described by Bárány in 1921 [1]. Dix and Hallpike established the clinical concept of the condition in 1952, which remains valid to date [2]. The concept of BPPV has been recently expanded to include a syndrome with positional nystagmus caused by free-floating particles, which may be otoconia detached from the otolith maculae [3]. The features of positional nystagmus depend on the location and status of these particles. When the particles exist freely in the lateral semicircular canal, paroxysmal direction-changing geotropic nystagmus (canalolithiasis type) is evoked, and when the particles adhere to the cupula, persistent direction-changing apogeotropic nystagmus (cupulolithiasis type) is evoked [3].

Shigeno et al. were the first to report cases of persistent direction-changing geotropic nystagmus that could not be explained by the existence of free-floating particles [4]. In these cases, nystagmus was present in the supine position but disappeared when the head was turned 30° to one side (the neutral point). No nystagmus was observed when the horizontal canal was placed on a horizontal plane. All of the features of these cases were identical to those of cupulolithiasis of the lateral semicircular canal, except for the direction of the positional nystagmus. Persistent direction-changing apogeotropic nystagmus in cupulolithiasis generates gravitational deviation of the cupula; thus, it was suggested that the persistent direction-changing geotropic nystagmus was evoked by antigravitational deviation of the cupula in the lateral semicircular canal. Shigeno et al. named this condition light cupula.

The clinical features of positional nystagmus in cases of light cupula have been described by several authors [5–11]. To the best of our knowledge, previously reported cases of positional nystagmus in patients with light cupula have spontaneously resolved within a few days [8–10]. Here, we report the case of a patient with positional nystagmus of the light cupula type that lasted for more than 6 months after the onset of sudden deafness with vertigo; the patient's symptoms were eventually resolved by plugging surgery.

## 2. Case Report

**Figure 1:**
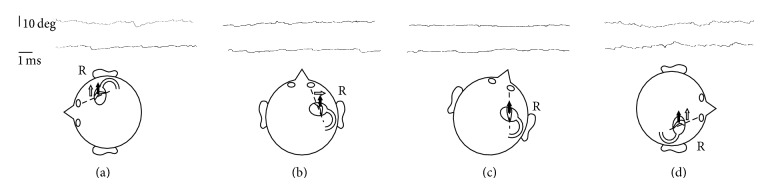
Electronystagmogram before surgery. Upper waves indicate eye position since turning head position. Lower waves indicate those one minute after maintaining the head position. The schemas indicate head positions and direction of copular deviations (outlined arrows). Filled arrows indicate direction of antigravitational vector and dotted lines indicate neutral position of cupula. (a) Left side down position. Antigravitational force deviates cupula of the right ear to the ampullofugal direction thus left beating nystagmus was observed. (b) Supine position. As the long axis of cupula is constitutionally out of alignment with anterior-posterior axis, cupula deviates to the ampullofugal direction and left beating nystagmus was also observed. (c) Approximately 30 degrees turn to right position. The long axis of cupula conforms with the antigravitational vector; thus cupula remains stationary and any nystagmus was not observed (neutral point). (d) Right side down position. Antigravitational force deviates cupula to the ampullopetal direction; thus right beating nystagmus was observed.

**Figure 2:**
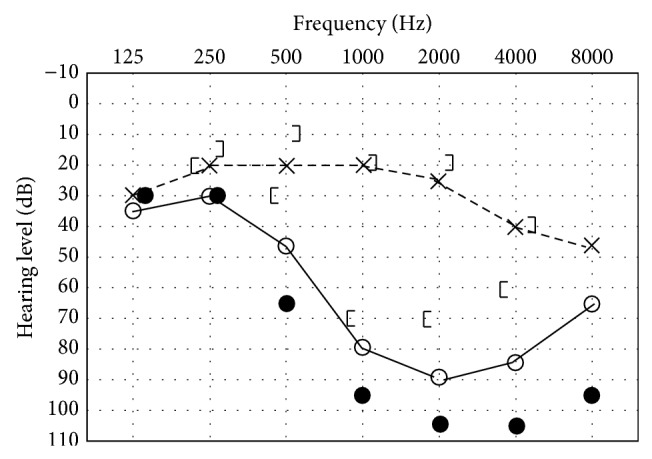
Audiogram before and after surgery. Filled circles indicate hearing level in the right ear after surgery.

A 67-year-old man who suffered from spontaneous vertigo with severe hearing loss in the right ear was diagnosed with idiopathic sudden hearing loss with vertigo and treated with an intravenous steroid by a local otolaryngologist. Although his hearing loss persisted, the patient's vertiginous sensation only lasted for a few days, and subsequent dizziness lasted for a few weeks. Three months after the onset of sudden hearing loss, he complained of a vertiginous sensation when in a right side down position. He was treated with an antivertiginous drug (Difenidol Hydrochloride 75 mg/day) by the local otolaryngologist, but his positional vertigo did not improve. The patient visited our clinic 8 months after the first onset of positional vertigo. He did not have any nystagmus in a sitting position, but a persistent direction-changing geotropic nystagmus was observed (Figure 1). A leftward beating nystagmus was observed when he was in a supine position, and this resolved when his head was turned approximately 30° to the right. A pure tone audiogram revealed high-frequency sensorineural hearing loss in the right ear (Figure 2). Monothermal (20°C) caloric testing revealed severe dysfunction on the right ear (maximum slow phase eye velocity was 26 degrees/second and 3 degrees/second on the left ear and the right ear, resp.). There were no cerebellar findings, and intracranial magnetic resonance imaging did not reveal any abnormal lesions. Based on the features of his nystagmus, he was diagnosed with light cupula of the right lateral semicircular canal. Despite continuous administration of the antivertiginous drug, positional vertigo and nystagmus did not improve. He also had difficulty with activities of daily living owing to the sensation of vertigo. After informed consent was obtained, plugging surgery of the right lateral semicircular canal was performed 11 months after the onset of positional vertigo. The surgical procedure used has been reported previously [10]. He complained of a spontaneous vertiginous sensation for 2 days but did not report any vertiginous sensation 1 month after surgery and was able to perform activities of daily living normally. His audiogram after surgery was not different to that before surgery (Figure 2). Postoperative caloric testing showed no response on the right ear even with ice water stimulation.

## 3. Discussion

The mechanisms of the antigravitational deviation of the cupula involved in light cupula are controversial. In summary, one mechanism that may be involved is a decrease in mass density due to an increase in ethanol concentration in the cupula after alcohol ingestion [11]. A second potential mechanism is an increased concentration of macromolecules in the endolymph, leading to a decrease in the relative density of the cupula [8, 9]. However, neither of these theories can explain why light cupula occurs in the lateral semicircular canal only [6]. A third potential mechanism is the adherence of light particles to the cupula, similar to the adherence of heavy particles that occurs in cupulolithiasis [8, 9]. The favorable prognosis of positional nystagmus in cases of light cupula can be explained by detachment of lightweight debris from the cupula. Therefore, most authors agree with this theory.

Our patient experienced positional nystagmus of the light cupula type for more than 6 months; thus, we suspected that the cause of this condition was an irreversible morphological change in the cupula due to preceding idiopathic sudden hearing loss rather than particle adherence. Inagaki et al. suggested that vestibular dysfunction in patients with idiopathic sudden hearing loss originates from morphological changes in the cupula, because the density of vestibular sensory cells is almost normal in cases with vestibular dysfunction [12]. To the best of our knowledge, morphological changes in the cupula have never been reported in studies on the human temporal bone. According to animal models, the cupula shrinks into the inner ear after gentamicin injection and enlarges after disruption of the membranous labyrinth [13, 14]. When the cupula becomes enlarged and the mass remains constant, its relative density should decrease.

Since the top of cupula attaches to the ampullar wall, an occluded semicircular canal creates a closed space between the cupula and the occluded site. Although the space is surrounded by soft tissue as membranous labyrinth and cupula, the animal study confirmed that this space acts as a solid component and inhibits the compression or expansion of the endolymph; therefore the cupula becomes fixed [15, 16]. In other words, successful plugging surgery requires a closed fluid-filled space between the cupula and the occluded site. When the cupula shrinks, the top part detaches from the ampullar wall and a closed space cannot be formed. On the other hand, an enlarged cupula maintains a closed space; thus, plugging surgery can produce successful results. As mentioned above, we speculated that disruption of the membranous labyrinth with idiopathic sudden hearing loss and consequent enlargement of the cupula caused the light cupula type of positional vertigo observed in the current case.
